# The Combination of SOFA Score and Urinary NGAL May Be an Effective Predictor for Ventilator Dependence among Critically Ill Surgical Patients: A Pilot Study

**DOI:** 10.3390/diagnostics11071186

**Published:** 2021-06-30

**Authors:** Hsin-I Tsai, Yu-Chieh Lu, Hao-Wei Kou, Heng-Yuan Hsu, Song-Fong Huang, Chun-Wei Huang, Chao-Wei Lee

**Affiliations:** 1 Linkou Medical Center, Department of Anesthesiology, Chang Gung Memorial Hospital, Taoyuan 333, Taiwan; tsaic@hotmail.com; 2 College of Medicine, Chang Gung University, Taoyuan 333, Taiwan; 3 Graduate Institute of Clinical Medical Sciences, College of Medicine, Chang Gung University, Taoyuan 333, Taiwan; 4 Linkou Medical Center, Department of Surgery, Chang Gung Memorial Hospital, Taoyuan 333, Taiwan; asey56@gmail.com; 5 Linkou Medical Center, Division of General Surgery, Department of Surgery, Chang Gung Memorial Hospital, Taoyuan 333, Taiwan; jeffreykou0417@gmail.com; 6 Department of Surgery, New Taipei Municipal Tu-Cheng Hospital (Built and Operated by Chang Gung Medical Foundation), New Taipei City 236017, Taiwan; smallredshoe@cgmh.org.tw (H.-Y.H.); woodyduck1983@gmail.com (S.-F.H.); n740227@cgmh.org.tw (C.-W.H.)

**Keywords:** NGAL, ventilator dependence, SOFA score, critically ill

## Abstract

Background: Ventilator dependence (VD) has been considered as a serious complication in critically ill patients in the intensive care unit (ICU). Acute kidney injury (AKI) is associated with VD as a result of lung–kidney interaction. The aim of our study was to investigate novel biomarkers in predicting ventilator dependence in critically ill surgical patients. Methods: Patients who were admitted to surgical ICU were enrolled and their serum and urine samples were collected. Novel biomarkers including gelatinase-associated lipocalin (NGAL), calprotectin, kidney injury molecule-1 (KIM-1), cystatin C, and growth differentiation factor 15 (GDF-15) were analyzed and correlated with clinical outcome. Results: A total of 33 patients were enrolled and analyzed. The majority of them received abdominal surgery prior to ICU admission. Thirteen patients were classified into the VD group, while the remaining 20 were in a non-ventilator dependence group (nVD). Statistical analysis demonstrated that the following were significantly higher in the VD group than in the nVD group: serum NGAL (420.25 ± 45.18 ng/mL vs. 314.68 ± 38.12 ng/mL, *p*-value 0.036), urinary NGAL (420.87 ± 41.08 ng/mL vs. 250.84 ± 39.45 ng/mL, *p*-value 0.002), SOFA score (11.3 ± 1.5 vs. 5.6 ± 0.7, *p*-value 0.001), and APACHE II score (23.2 ± 2.6 vs. 13.6 ± 0.8, *p*-value 0.001). The area under the ROC curve (AUROC) of urinary NGAL for VD was 0.808. The combination of urinary NGAL and SOFA score could further increase AUROC for VD to 0.835. Conclusions: The current study demonstrated the predictive capability of urinary NGAL for ventilator dependence among critically ill surgical patients. When combined with SOFA score, the predictive ability was further augmented. Further large-scale studies are warranted to validate our findings.

## 1. Background

Postoperatively, pulmonary complications are one of the main issues in the intensive critical unit (ICU) [1]. Adequate oxygenation for tissue perfusion is of major importance for post-surgical recovery. Some patients, in particular those who receive abdominal surgery, may be subject to hypoxemia or develop acute respiratory failure secondary to the state of severe pulmonary restriction. Perioperative factors such as patients’ existing co-morbidities, ventilator settings during general anesthesia, and postoperative pain management all play a role in the decline of respiratory function, leading to a decrease in pulmonary volume, diaphragm dysfunction, and atelectasis [2]. Mechanical ventilation (MV) is believed to assist patients to go through a critical time and minimize pulmonary complications postoperatively, such as atelectasis or pneumonia; however, prolonged use of invasive MV may be associated with increased risks of pulmonary disease and in-hospital mortality. Reviewing pre-existing literature, the definition of prolonged MV has been variously determined based on the duration of MV (ranging from two to twenty-nine days) and the mode of MV (invasive or non-invasive) [3,4]. That said, the optimal length of invasive MV is not determined and there exists various suggestions according to the respective criteria.

Ventilator dependence has long been considered as a serious complication in critically ill patients in the ICU [5]. Ventilator-dependent patients appear to endure a higher rate of kidney injury requiring hemodialysis, longer ICU and hospital stay, and significantly higher in-hospital mortality than those who do not require MV [6]. To recognize the timing for successful extubation from MV is therefore of utter importance for the intensivists [5,7]. Clinically, conservative strategies consisting of weaning protocols and spontaneous breathing trials have been practiced prior to extubation [8], nonetheless, a failure to wean from MV still occurs [7,9]. Recent literature has shown that acute kidney injury (AKI) is also associated with ventilator dependence in critically ill patients in the ICU, possibly as a result of lung–kidney interaction [10,11]. Several major mechanisms to explain the underlying pathophysiology were proposed. Inflammatory mediators were increasingly produced but decreasingly cleared in patients with impaired renal function leading to detrimental effects on their lungs [12]. Others suggested the risk of pulmonary infection was increased secondary to impaired immune function as a result of kidney injury [13]. In addition, fluid overload followed by oliguria in acute kidney injury could lead to respiratory failure [14].

As novel biomarkers are discovered to timely predict acute kidney injury, such biological markers may offer an opportunity to predict ventilator dependence in critically ill surgical patients [15]. Traditionally, serum creatinine (Cr) level and blood urea nitrogen (BUN) rise when half of the kidney function is impaired [16], but in patients with sarcopenia such an increase may not be observed despite a substantial kidney decline [17,18]. Serum Cr and BUN do not differentiate kidney damage and are classified according to different guidelines and criteria, which could be confusing sometimes [19]. Since lung–kidney interaction plays a major role in critically ill patients in the ICU, we hypothesized that patients with acute kidney injury are at an elevated risk for respiratory failure and, thus, difficulty weaning from mechanical ventilation. The aim of our study was to employ biomarkers in association with acute kidney injury, including neutrophil gelatinase-associated lipocalin (NGAL), calprotectin, kidney injury molecule-1 (KIM-1), cystatin C, and growth differentiation factor 15 (GDF-15), along with SOFA and APACHE scores in predicting ventilator dependence in critically ill surgical patients.

## 2. Material and Methods

### 2.1. Data Source and Patient Population

From November 2014 to June 2015, patients admitted to the surgical intensive care unit of Chang Gung Memorial Hospital (CGMH) were prospectively enrolled. To avoid postoperative care bias, only patients in 10-bed surgical ICU in the Department of General Surgery of CGMH were included. In our hospital, surgical patients were admitted to surgical ICU by the following indications: the presence of acute postoperative respiratory distress, hemodynamic instability of any etiology, major postoperative complications requiring invasive monitoring, life-threatening co-morbidities such as acute liver failure, gastrointestinal bleeding, or other concerns acknowledged as per surgeon or anesthesiologist. Patients who were less than 20 years of age, had a history of declined kidney function, required routine dialysis intervention, or received renal transplantation were excluded from the study. Patients who had inflammatory bowel disease were also excluded. Declined kidney function was defined as chronic kidney disease for more than 3 months. A total of 242 patients were informed. After excluding those who refused to participate or without legal consent and whose blood/urine specimens were not collected within 24 h of ICU admission, 33 patients were eventually enrolled. Ventilator dependence was defined as: (1) difficulty weaning from invasive mechanical ventilation with a prolonged use for more than 14 days, or (2) death under mechanical ventilation use. The patients were grouped into a ventilator dependence group (VD) and non-ventilator dependence group (nVD). Upon ICU admission, patients were evaluated and recorded with the Acute Physiology and Chronic Health Evaluation (APACHE II) and the Sequential Organ Failure Assessment (SOFA) scores according to the value of clinical data and basic vital signs, such as temperature, heart rate (HR), respiratory rate (RR), mean arterial blood pressure (MAP), Glasgow Coma Scale (GCS), Acid-Base condition (pH level), partial pressure of oxygen (PaO_2_), fraction of inspired oxygen (FiO_2_), serum platelet count (PLT), serum sodium (Na), serum potassium (K), serum white blood count, serum hematocrit (Hct), serum bilirubin, serum creatinine (Cr), and urine output over the following 24 h. The patients were closely observed during their stay in surgical ICU. The diagnosis of sepsis was defined by the criteria established by the 45th Critical Care Congress in 2016, also known as Sepsis-3 [20,21]. The present study was approved by the Institutional Review Board of Chang Gung Memorial Hospital (CGMH IRB103-2722A3) and conducted according to the Declaration of Helsinki.

### 2.2. Blood Sampling and Assays

We obtained the blood and urine samples from the enrolled patients once admitted to our surgical ICU. Blood samples were centrifuged at 1500× *g* for 10 min, while urine samples were centrifuged at 500× *g* for 10 min; both were aliquoted and stored at −80 °C for batch analysis. Serum and urinary gelatinase-associated lipocalin (NGAL), calprotectin, kidney injury molecule-1 (KIM-1), cystatin C, and growth differentiation factor 15 (GDF-15) were measured using an enzyme-linked immunosorbent assay (ELISA) kit (DuoSet ELISA, R&D Systems; Minneapolis, MN, USA) [13,14,16,17,18,19,22]. The dilution ratios for NGAL, calprotectin, KIM-1, cystatin C, and GDF-15 were 1:100, 1:1, 1:100, 1:400, and 1:100, respectively.

### 2.3. Statistical Analysis 

The statistical information, including patient general data, clinical indicators, and serum/urinary biomarkers, were collected accurately. Data were analyzed statistically using IBM SPSS v. 25 [23] and R software [24]. The continuous variable data were tested using Mann–Whitney U test and presented by mean ± standard error of mean (Mean ± SEM). The categorical variables data were compared using Fisher’s test or Pearson’s chi-square test and shown as absolute frequency and percentages. For the prediction of ventilator dependence, we performed logistic regression and receiver operating characteristic of variable factors and biomarker concentration. For all statistical tests, *p*-value < 0.05 was considered to be statistically significant.

## 3. Result

### 3.1. Study Population

**Figure 1:**
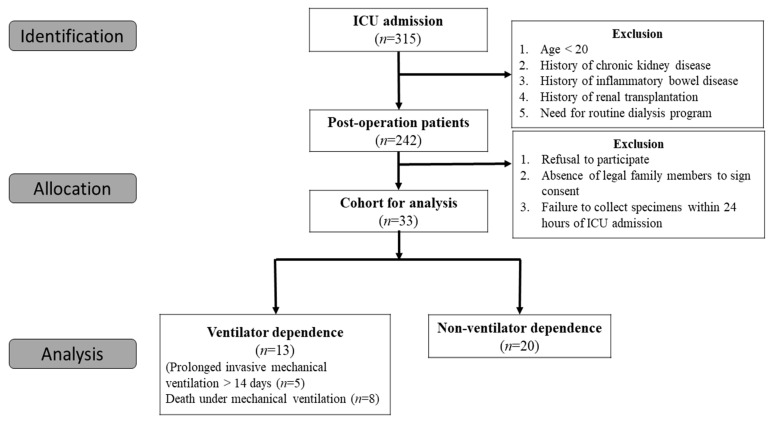
Flow chart of the enrolled patients.

Figure 1 is the study flowchart of the current research. After excluding ineligible patients, a total of 33 patients were enrolled. Among them, thirteen patients were defined as ventilator dependent (VD), with five patients under prolonged MV for more than fourteen days and eight deaths under MV.

### 3.2. Patient Characteristics

Table 1 summarizes the patient demographics. Around 60% of the patients were male and the mean age was 66.3 years. More than 80% of the patients received major intra-abdominal operations prior to ICU admission. Among the patients enrolled, thirteen (39.4%) were categorized into the VD group and twenty (60.6%) into the nVD group. The mean duration of invasive MV was 8.85 days and the mean ICU stay was 12.64 days. The mean SOFA score was 7.848 and mean APACHE II score was 17.364 upon ICU arrival. Fifteen (45.45%) patients deceased during this hospitalization. 

**Figure 2:**
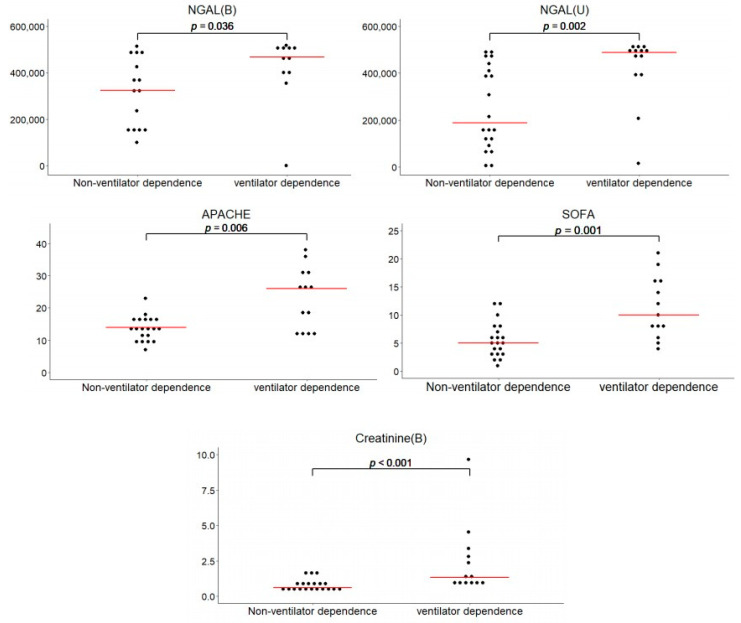
Serum and urinary levels of NGAL, SOFA score, APACHE II score, and serum creatinine in relation to ventilator dependence. The levels are represented as scatter dot plots, and the arithmetic means of the tested parameters are indicated by a line. APACHE, Acute Physiology and Chronic Health Evaluation; NGAL, neutrophil gelatinase-associated lipocalin; SOFA, Sequential Organ Failure Assessment.

Table 2 demonstrates the differential features between the VD and nVD groups. Compared with patients in the nVD group, the levels of serum creatinine, APACHEII score, and SOFA score were significantly higher in the VD group (*p* < 0.001, 0.006 and 0.001, respectively) (Figure 2). The duration of invasive MV, ICU stay, and hospital stay were also significantly longer in the VD group (*p* = 0.014, 0.036 and 0.024, respectively). There was no difference in the baseline estimated glomerular filtration rate (eGFR), amount of urine output, and subsequent dialysis requirement. Ventilator dependence was found to considerably increase the rate of in-hospital mortality in the current study (84.62% in the VD group vs. 20% in the nVD group, *p* < 0.001).

### 3.3. Analysis of Biomarkers Regarding Ventilator Dependence

The levels of five common biomarkers related to kidney injury are summarized in Table 3. Both the serum and urinary levels of NGAL, calprotectin, KIM-1, cystatin C and GDF-15 were analyzed. The levels of serum and urinary NGAL were significantly higher in the VD group than in the nVD group (serum NGAL, 420.25 ± 45.18 ng/mL vs. 314.68 ± 41.08 ng/mL, *p* = 0.036; urinary NGAL, 420.87 ± 38.12 ng/mL vs. 250.84 ± 39.45, *p* = 0.002; Figure 2). There was no significant difference in the remaining four biomarkers between the VD and nVD groups.

### 3.4. Performance of Biomarkers in Predicting Ventilator Dependence

**Figure 3:**
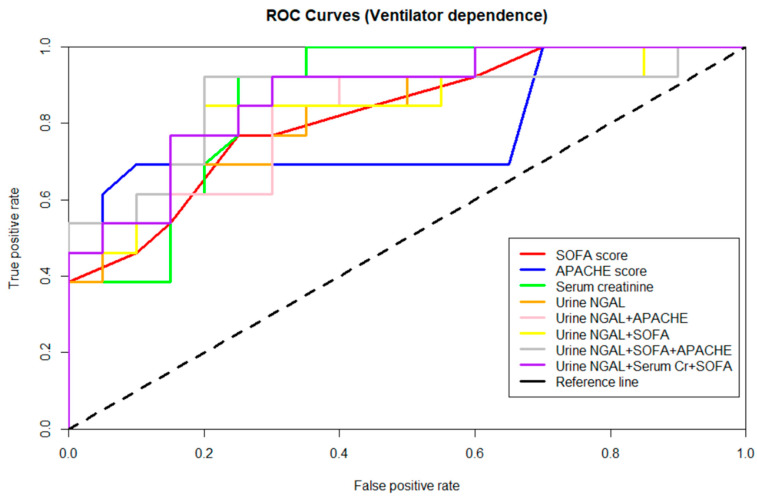
**Performance of biomarkers.** ROC curves of clinical variables and biomarkers in predicting ventilator dependence. APACHE, Acute Physiology and Chronic Health Evaluation; NGAL, neutrophil gelatinase-associated lipocalin; SOFA, Sequential Organ Failure Assessment.

To predict the possibility of ventilator dependence among critically ill surgical patients, receiver operating characteristic (ROC) curves were employed and various clinical parameters as well as biomarkers were analyzed. For biomarkers, the area under the ROC curve (AUROC) of serum NGAL alone in predicting ventilator dependence was 0.789, while that of urinary NGAL alone was 0.808 (Figure 3 and Table 4). Among the clinical parameters, the AUROC of serum creatinine in predicting VD was 0.867. Meanwhile, the AUROC of APACHEII score and SOFA score was 0.783 and 0.821, respectively. A logit model of the logistic regression incorporating these significant variables was established to yield a single score to predict ventilator dependence. The AUROC for VD was 0.835 when both urinary NGAL and SOFA score were combined. The incorporation of urinary NGAL, SOFA score, and serum creatinine could further elevate the AUROC to 0.873. The AUROC of various biomarkers and clinical parameters are summarized in Table 4.

## 4. Discussion

Ventilator dependence remains one of the unresolved challenges in the intensive care unit, and prolonged mechanical ventilation is believed to increase the rate of in-hospital mortality. Since acute kidney injury has been demonstrated to induce ventilator dependence in critically ill patients by lung–kidney interaction, ventilator protocols guided by kidney function may thus serve as a promising strategy to improve clinical outcomes in the ICU [25]. Creatinine is commonly employed as a surrogate for renal function and is adopted in various guidelines to define acute kidney injury. It has also been demonstrated to be a potential prognostic factor for ventilator dependence [26]. For example, the measured creatinine clearance has been reported to be a significant predictor for ventilator dependence [27]. Studies have shown that the creatinine height index, a part of lean muscle mass, was a strong predictor for successful weaning and survival in patients under prolonged mechanical ventilation [28]. However, the level of serum creatinine may be misleading in patients with chronic kidney disease or end-stage renal disease under dialysis. In addition, as mentioned above, the elevation of serum creatinine upon renal impairment may not be observed in patients with sarcopenia, which is a common phenomenon among critically ill surgical patients [29]. Furthermore, the rise of serum creatinine is usually delayed upon renal injury, and it did not provide insight into the location, severity, and pathological process of kidney dysfunction [30]. Fortunately, novel biomarkers predictive of renal dysfunction are emerging, and they exhibit the ability to detect acute kidney injury prior to the escalation of serum creatinine [31,32]. 

For instance, neutrophil gelatinase-associated lipocalin(NGAL), a 25-kDa protein belonged to the family of lipocalins, is a well-known inflammatory marker frequently elevated in structural renal tubular damage. It rises as early as 3 h after inflammatory or ischemic renal injury and peaks at about 6 to 12 h [26,33,34]. A systemic review and meta-analysis reported a composite AUROC 0.72 for early prediction of AKI after cardiac surgery in adults [35]. As a result, NGAL could be a promising biomarker for the early detection of AKI in critically ill patients. On the other hand, however, the predictive capability of NGAL and other novel biomarkers for ventilator dependence has not been determined. The current study, by prospectively analyzing serum and urinary biomarkers from patients in the surgical ICU of a tertiary care center, is one of the first studies in the English literature to demonstrate the ability of renal markers to predict ventilator dependence. Both serum and urinary NGAL are good predictors for ventilator dependence with favorable sensitivity and specificity. Previous studies also reported that the elevation of NGAL levels was related to significantly longer intubation after pediatric cardiac surgery with an estimated AUROC of 0.9 upon ICU admission [36]. As mentioned above, since lung–kidney interaction may induce ventilator dependence in critically ill patients and the release of inflammatory mediators by ventilator-induced kidney injury is also implicative in the destruction of the kidney, the elevated levels of NGAL detected in the VD group could be reasonably explained. 

By comparing the performance of NGAL, GDF-15, cystatin C, KIM-1 and calprotectin, in combination with other clinical parameters, in predicting ventilator dependence in critically ill surgical patients, the current study also demonstrated equivalent AUROC for VD between the combination of urine NGAL + SOFA score + APACHE II score and serum creatinine alone. Our finding indicates the possibility of real-time assessment of the renal-protective and patient-specific mechanical ventilation strategy. The incorporation of these biomarkers into our routine weaning evaluation could provide patient-specific ventilator setting, prevent further kidney damage, and decrease in-hospital mortality.

Despite remarkable results, the current study still has several limitations. First, due to the emergent setting and the requirement for informed consent, the patient population was rather small. Second, the collections of serum and urine samples were limited to a single time point. Third, despite urinary NGAL having a higher specificity than serum creatinine, the AUROC of serum creatinine was still better than that of other biomarkers. We believe this may be due to our limited patient number and the exclusion of patients with chronic kidney disease or routine dialysis. Fourth, the current study only revealed the predictive capability of novel biomarkers for VD. The exact relationships or mechanisms underlying this finding remain undetermined. As a result, further prospective, large-scale studies examining biomarkers at different time points are necessary to validate and explain our findings.

## 5. Conclusions

In conclusion, the current study demonstrated the predictive capability of urinary NGAL for ventilator dependence among critically ill surgical patients. When combined with SOFA/APACHE II scores, the predictive ability was further augmented. We believe the incorporation of these promising biomarkers into our ICU routine may help clinicians detect the onset of AKI, guide the course of treatment, and predict outcomes at an earlier stage. Further large-scale studies are warranted to validate our findings. 

## Figures and Tables

**Table 1:** Clinical characteristics of patients at ICU admission (*n* = 33).

Categorical Variables		No.	%
Gender	Male	20	61
	Female	13	39
Comorbidity	Chronic lung disease	3	9.1
	Hypertension	10	30.3
	Liver cirrhosis	4	12.1
	Cardiovascular disease	5	15.2
	Diabetes mellitus	5	15.1
	Cerebrovascular disease	3	9.1
	Malignancy	18	54.5
Type of surgery	Hepatobiliary surgery	6	18.2
	Gastrointestinal surgery	21	63.6
	Others	2	6.1
	Without surgery	4	12.1
Ventilator dependence	Yes	13	39.4
	No	20	60.6
Dialysis	Yes	2	6.06
	No	31	93.94
In-hospital mortality	Yes	15	45.45
	No	18	54.55
3 months mortality	Yes	15	45.45
	No	18	54.55
6 months mortality	Yes	16	48.48
	No	17	51.52
12 months mortality	Yes	16	50
	No	16	50
**Continuous Variables**	**Mean**	**SE ^a^**	
Age (years)	66.33	2.56	
BMI (kg/m^2^)	23.58	0.62	
SOFA score ^b^	7.848	0.874	
APACHE II score ^c^	17.364	1.401	
MV ^d^ (days)	8.85	3.53	
ICU ^e^ stay (days)	12.64	4.41	
Hospital stay (days)	39.09	4.38	
Baseline Egfr ^f^ (mL/min)	107.1842	6.52	
Creatinine (mg/dL)	1.426	0.303	
eGFR (mL/min)	80.38424	8.92	
Urine output(mL/day)	1548.788	190.871	
Albumin (g/dL)	2.733	0.100	
White blood cell count (1000/μL)	14.352	1.545	
CRP ^g^ (mg/L)	123.329	18.414	
Procalcitonin (ng/mL)	33.761	12.234	

^a^ standard error; ^b^ sequential organ failure assessment; ^c^ acute physiology and chronic health evaluation; ^d^ mechanical ventilation; ^e^ intensive care unit; ^f^ estimated glomerular filtration rate; ^g^ C-reactive protein.

**Table 2:** Clinical characteristics of patients categorized by ventilator dependence (*n* = 33).

Variables	Ventilator Dependence (*n* = 13)	Non-Ventilator Dependence (*n* = 20)	*p*-Value
	Mean ± SEM ^a^	Mean ± SEM ^a^	
Age (years)	63.23 ± 4.54	68.35 ± 3.03	0.353
BMI (kg/m^2^)	22.45 ± 0.933	24.31 ± 0.798	0.169
APACHE II ^b^ score	23.154 ± 2.641	13.600 ± 0.835	0.006
SOFA ^c^ score	11.308 ± 1.521	5.600 ± 0.705	0.001
MV ^d^ (days)	19.31 ± 8.295	2.05 ± 0.478	0.014
ICU ^e^ stays (days)	27.62 ± 10.005	2.90 ± 0.447	0.036
Hospital stays (days)	52.31 ± 8.860	30.50 ± 3.335	0.024
Baseline eGFR ^f^ (mL/min)	103.4254 ± 9.731097	109.63 ± 8.86	0.65
Creatinine (mg/dL)	2.412 ± 0.684	0.786 ± 0.095	<0.001
eGFR upon ICU admission ^f^ (mL/min)	49.875 ± 8.073	100.215 ± 11.911	0.001
Urine output (mL/day)	1198.308 ± 288.885	1776.600 ± 245.046	0.235
White blood cell count (1000/μL)	18.654 ± 3.026	11.555 ± 1.354	0.027
CRP ^g^ (mg/L)	146.519 ± 23.598	101.796 ± 27.505	0.054
Procalcitonin (ng/mL)	40.640 ± 17.401	24.818 ± 17.347	0.410
	**No. (%)**	**No. (%)**	***p*-Value**
Sepsis			0.002
Yes	13 (100)	9 (45.0)	
No	0 (0)	11 (55.0)	
In-hospital mortality			<0.001
Yes	11 (84.62)	4 (20.0)	
No	2 (15.38)	16 (80.0)	
Dialysis			0.148
Yes	2 (15.38)	0 (0)	
No	11 (84.62)	20 (100)	

^a^ standard error; ^b^ acute physiology and chronic health evaluation; ^c^ sequential organ failure assessment; ^d^ mechanical ventilation; ^e^ intensive care unit; ^f^ estimated glomerular filtration rate; ^g^ C-reactive protein.

**Table 3:** Serum and urinary biomarkers of patients categorized by weaning outcome (*n* = 33).

Variables	Ventilator Dependence (*n* = 13)		Non-Ventilator Dependence (*n* = 20)		*p*-Value
	Mean	SEM ^a^	Mean	SEM ^a^	
Serum					
NGAL (ng/mL)	420.25	45.18	314.68	38.12	0.036
Calprotectin (pg/mL)	1026.90	386.21	407.06	185.17	0.097
KIM-1 (pg/mL)	984.08	516.69	572.57	253.75	0.760
Cystatin C (pg/mL)	1323.27	285.35	838.96	118.19	0.069
GDF-15 (ng/mL)	6.32	2.61	11.09	2.71	0.097
Urine					
NGAL (ng/mL)	420.87	41.08	250.84	39.45	0.002
Calprotectin (pg/mL)	970.74	489.55	775.46	295.20	0.221
KIM-1 (pg/mL)	8433.73	2276.40	7689.04	1802.37	0.758
Cystatin C (ng/mL)	378.18	141.11	147.96	45.50	0.477
GDF-15 (ng/mL)	24.56	4.68	30.30	2.85	0.281

^a^ Standard error of mean.

**Table 4:** Area under the ROC curve (AUROC) in predicting ventilator dependence (*n* = 33).

Variables ^a^	Prediction of Ventilator Dependence			*p* Value
	AUROC	Cut-Off Value	Sensitivity/Specificity (%)	
Serum				
NGAL (ng/mL)	0.789	398.4	81.82/66.67	0.036
Calprotectin (pg/mL)	0.694	150.8	81.82/66.67	0.097
KIM-1 (pg/mL)	0.522			0.87
Cystatin C (pg/mL)	0.715	1008.29	81.82/73.33	0.065
GDF-15 (ng/mL)	0.389			0.414
Creatinine (mg/dL)	0.867	0.94	92.31/71.4	0.001
CRP (mg/L)	0.720	89.03	84.62/64.29	0.052
Urine				
NGAL (ng/mL)	0.808	465.2	69.23/85	0.003
Calprotectin (pg/mL)	0.392			0.312
KIM-1 (pg/mL)	0.579			0.46
Cystatin C (ng/mL)	0.579			0.46
GDF-15 (ng/mL)	0.417			0.436
APACHE II score	0.783	18	69.23/90	0.007
SOFA score	0.821	8	76.92/75	0.002
Urine NGAL + APACHE score	0.827			0.002
Urine NGAL + SOFA score	0.835			0.001
Urine NGAL + APACHE score + SOFA score	0.865			<0.001
Urine NGAL + Serum Cr + SOFA score	0.873			<0.001

^a^ standard error.

## Data Availability

All data generated or analyzed during the study are included in this published article. Raw data may be requested from the authors with the permission of the institution.

## Abbreviations

AKI	acute kidney injury
APACHE	Acute Physiology and Chronic Health Evaluation
AUROC	area under the ROC curve
CGMH	Chang Gung Memorial Hospital
eGFR	estimated glomerular filtration rate
ELISA	enzyme-linked immunosorbent assay
FiO_2_	fraction of inspired oxygen
GCS	Glasgow coma scale
GDF-15	growth differentiation factor 15
ICU	intensive care unit
IRB	institutional review board
KIM-1	kidneyinjury molecule-1
MAP	mean arterial blood pressure
MV	mechanical ventilation
NGAL	neutrophil gelatinase-associated lipocalin
nVD	non-ventilator dependence
PaO_2_	partial pressure of oxygen
ROC curve	receiver operating characteristic curve
SOFA	Sequential Organ Failure Assessment
VD	ventilator dependence
